# Exercise Therapies for Parkinson's Disease: A Systematic Review and Meta-Analysis

**DOI:** 10.1155/2020/2565320

**Published:** 2020-09-08

**Authors:** Hyun-young Choi, Ki-Ho Cho, Chul Jin, JiEun Lee, Tae-Hun Kim, Woo-Sang Jung, Sang-Kwan Moon, Chang-Nam Ko, Seung-Yeon Cho, Chan-Yong Jeon, Tae Young Choi, Myeong Soo Lee, Sang-Ho Lee, Eun Kyoung Chung, Seungwon Kwon

**Affiliations:** ^1^Department of Korean Medicine Cardiology and Neurology, Graduate School, Kyung Hee University, Seoul 02447, Republic of Korea; ^2^Department of Cardiology and Neurology, College of Korean Medicine, Kyung Hee University, Seoul 02447, Republic of Korea; ^3^Korean Medicine Clinical Trial Center, Korean Medicine Hospital, Kyung Hee University, Seoul 02447, Republic of Korea; ^4^Department of Korean Internal Medicine, College of Korean Medicine, Gachon University, Seongnam 13120, Republic of Korea; ^5^Clinical Research Division, Korea Institute of Oriental Medicine, Daejeon 34054, Republic of Korea; ^6^Gangdong Mokhuri Oriental Medical Hospital, Department of Internal Medicine, Seoul 05316, Republic of Korea; ^7^Division of Clinical Pharmacy, Department of Pharmacy, College of Pharmacy, Kyung Hee University, Seoul 02447, Republic of Korea

## Abstract

Recently, rehabilitative exercise therapies have been described as an important method of overcoming the limitations of the conventional therapies for Parkinson's disease. The present study aimed to evaluate efficacy and safety of exercise therapies for Parkinson's disease. Randomized controlled trials that evaluated exercise therapies in patients with Parkinson's disease until December 2016 were searched for in five electronic databases: PubMed, CENTRAL, EMBASE, OASIS, and CNKI. Eighteen studies (1,144 patients) were included. The overall methodological quality was not high. Patients who underwent exercise therapies exhibited statistically significant improvements in the total UPDRS, UPDRS II and III, Berg Balance Scale, preferred walking speed, and Timed Up and Go Test compared to patients who underwent nonexercise therapies. In comparison to patients who performed regular activity, patients who underwent exercise therapies exhibited statistically significant improvements in the total UPDRS, UPDRS II, and UPDRS III. Exercise therapies were found to be relatively safe. Exercise therapies might promote improvements in the motor symptoms of Parkinson's disease. However, due to the small number of randomized controlled trials and methodological limitations, we are unable to draw concrete conclusions. Therefore, further studies with better designs will be needed.

## 1. Introduction

Parkinson's disease (PD) is a neurodegenerative neurological disease characterized by a decrease in dopaminergic neurons in the substantia nigra pars compacta (SNpc) and lowered dopamine concentrations in the basal ganglia [1]. Symptoms are divided into motor and nonmotor symptoms. Motor symptoms are characterized by bradykinesia, rigidity, resting tremor, and postural instability. There are also several nonmotor symptoms such as anosmia, sleep disorders, psychiatric symptoms, cognitive impairment, autonomic dysfunction, fatigue, and pain [2].

The motor symptoms of PD begin to appear in the early stage of the disease, leading to a decrease in the quality of life (QOL) [2, 3]. PD patients stay in hospital for about 1.45 times longer than healthy persons for about 2–14 days. Furthermore, they are more likely to be exposed to emergency situations such as falls [4]. The prevalence and incidence of PD has been increased gradually [5, 6]. According to the statistics up to 2016, 6.1 million patients suffer from PD globally [6].

In general, anti-Parkinsonian medications such as levodopa, dopamine agonists, monoamine oxidase type B inhibitors (MAOBIs), amantadine, and anticholinergics are administered as first-choice treatment. However, long-term use of dopaminergic medications could lead to adverse effects such as peak-dose dyskinesia, on-off phenomenon, and wearing off [7]. Surgical treatment such as thalamotomy, chemopallidectomy, and deep brain stimulation has been used to reduce the physiological changes of brain tissue caused by PD [8, 9]. However, it is expensive, it has high risk of side effects [10], and the possibility of reoperation cannot also be ruled out. Therefore, complementary therapies such as rehabilitation exercises could be considered in a long-term perspective. Previous studies suggested that rehabilitation exercise therapies could activate the central and peripheral nervous systems, thereby maximizing body function and slowing the progression of the disease [11].

Recently, there has been a growing interest in constructing rehabilitation strategies for PD patients in a comprehensive and diverse manner, one of which is exercise. According to animal studies, exercise therapies have neuroprotective effects and an inhibitory effect on the progression of PD or the restoration of the disease in animals. The neuroprotective effect of exercise on humans has not yet been clearly reported, but exercise therapy is most likely to be used in clinical practice [12].

The purpose of this systematic review and meta-analysis study was to investigate the effect and safety of exercise therapies on PD. To reflect the differences in exercise interventions used in each of the existing studies, we performed a meta-analysis by grouping them according to the nature of the exercise interventions.

## 2. Materials and Methods

### 2.1. Study Design

This study is a systematic review and meta-analysis to examine the effect and safety of exercise therapies on patients with PD.

### 2.2. Data Sources and Search Strategy

This study was carried out according to the PRISMA (Preferred Reporting Items for Systematic Reviews and Meta-Analyses) guidelines [13] and the Cochrane Handbook for Systematic Reviews of Interventions [14]. The systematic literature search was conducted using Pubmed (Medline), Excerpta Medica dataBASE (EMBASE), Cochrane Central Register of Controlled Trials (CENTRAL), the Oriental Medicine Advanced Searching Integrated System (OASIS), and Chinese medical databases (CNKI- Chinese Academic Journal). The articles reported until December 2016 were searched, and there was no language limitation. Various exercise terms and MeSH terms were used for searching. The search strategies used in each database are presented in Table 1.

### 2.3. Study Selection

The criteria for the selection of the literature were as follows: randomized controlled trials which evaluated the effect of walking training, strength or flexibility training, balancing training, and aerobic training on patients with PD. We excluded nonrandomized or uncontrolled trials, *in vivo* or *in vitro* studies, statistical studies, or protocol papers. In the case of duplicate documents, when more than two studies were available, the most recently reported or more complete literature was selected.

### 2.4. Type of Participants

Studies involving patients with PD were selected. UK Parkinson's Disease Society Brain Bank clinical diagnostic criteria was used as the PD diagnostic criteria [15]. There were no restrictions on sex, age, race, or disease duration. Patients with other diseases such as dementia, chronic medical illnesses, and atypical or secondary Parkinsonism were excluded.

### 2.5. Type of Interventions

Studies that used exercise therapy as an intervention for PD were included. We also included studies that used an auxiliary device for exercise such as a treadmill, but excluded studies in which the device was used as a core intervention, such as Nintendo or robot. There were no limitations on program content, such as exercise treatment methods, progress, frequency, duration, and intensity, but we only included studies which had the program of activity developed in detail. Qigong therapy in East Asian traditional medicine such as tai chi was not included neither.

In this study, the studies were classified according to each type of exercise treatment; meanwhile, they were classified as complex exercise when two types of exercises were used. The types of exercise are as follows: walking exercise either on a treadmill or on flat ground; balancing exercise, referring to the movement that shifts from one movement to another, holds a posture, and delays adjustment through physical cooperation; aerobic exercise, referring to the movement that involves stepping with a partner, tapping the ground, crossing the foot, or moving weight from one leg to the other; and dancing were included in this category; strength exercises, referring to training that prevents muscle weakness through the contraction of muscle fibers by external loads [16], and exercises that strengthen the quadriceps, hamstrings, gastrocnemius, and rectus abdominis muscles were included in this category.

### 2.6. Type of Comparisons

According to the type of the control group, the studies were divided into two categories. The first was for the conventional drug treatment (standard of care) and no exercise treatment. In this case, the experimental group performs the exercise therapy as an adjunctive intervention, and the control group continues the usual medication just as before the trial. Second, the control group performed a regular activity with a regular program. This program included any simple activity, physiotherapy, or cognitive activity without exercise, except for walking, balancing, aerobics, and strength training. The control group proceeded regularly in the same way during the trial process. However, studies with an active control group which performed similar intervention with the experimental group and studies with two different experimental groups (for example, walking vs. strength exercise) were excluded.

### 2.7. Type of Outcome Measures

The symptoms of PD were evaluated and divided into motor function, balance function, gait, quality of life (QOL), and general symptoms. In general, the Unified Parkinson's Disease Rating Scale (UPDRS) and the Movement Disorder Society Unified Parkinson's Disease Rating Scale (MDS-UPRDS [17, 18]) were used to evaluate the symptoms of Parkinson's disease. To assess motor function, the UPDRS and MDS-UPRDS part III were used, and total UPDRS and UPDRS part I/II were used to evaluate general symptoms in this study. The evaluation of balance function was carried out using the Berg Balance Scale (BBS) [19] and Timed Up and Go Test (TUGT) [20]. Gait function was assessed by the gait velocity and the 6-minute walk test. The gait velocity was evaluated in two ways: the preferred walking speed (m/s) and the fast walking speed (m/s). The preferred walking speed (m/s) was the measurement of the patient's most comfortable walking speed, while the fast walking speed (m/s) was the patient's maximum walking speed. Reported adverse effects were also extracted.

### 2.8. Data Extraction

Data extraction was conducted by two researchers (Hyun-young Choi and Seungwon Kwon), and an arbiter (Ki-Ho Cho) made the final decision if there was a disagreement between the 2 researchers. The first author, characteristics of the study (i.e., year, nation (English/Chinese), setting, and design), characteristics of participants (i.e., sex, sample size, Hoehn and Yahr scale (H&Y scale), disease duration, and medication), intervention details of the experimental and control groups, measured outcome, intergroup differences, and adverse events were extracted. If any of the abovementioned data was unclear, efforts were made to contact the authors of the study.

### 2.9. Quality Assessment in Individual Studies

Cochrane's risk of bias tool was used for the quality evaluation [21]. It is a tool for evaluating the bias of research included in the creation of systematic reviews and meta-analyses. It consists of 7 sections, and each was divided into “low risk of bias,” “unclear risk of bias,” and “high risk of bias.” The quality of the literature was assessed based on what is described in the literature. Risk of bias (ROB) assessment was conducted by two independent authors (Hyun-young Choi and Seungwon Kwon). In the event of a disagreement while extracting data or assessing the ROB, the third author (Ki-Ho Cho) resolved the discrepancy.

### 2.10. Synthesis of Data and Meta-Analysis

Meta-analysis was performed using Cochrane review manager software version 5.3 (RevMan 5.3). Based on the study design, a meta-analysis was conducted on the comparative study of the exercise with conventional drug treatment combination and conventional drug monotherapy groups. Separately, a meta-analysis was conducted on the comparative study of exercise and conventional drug treatment combination and on the regular activity and conventional drug treatment combination groups. The efficacy estimates were obtained from the relative risk (RR) for dichotomous variables and from the mean difference (MD) for continuous variables. A random effect model was used based on clinical heterogeneity between studies. The statistical significance of the effect estimates was verified based on the total effect test, 95% confidence interval (CI), and significance level of 5%. Meta-analysis was conducted by the classification of each outcome.

The Chi-square test and the Higgins *I*^2^ statistics were used to assess statistical heterogeneity. In the Chi-square test, it was verified that there was significant heterogeneity when the *p* value was less than 0.05 or the *I*^2^ value was greater than 50.

## 3. Results

### 3.1. Description of the Included Studies

**Figure 1:**
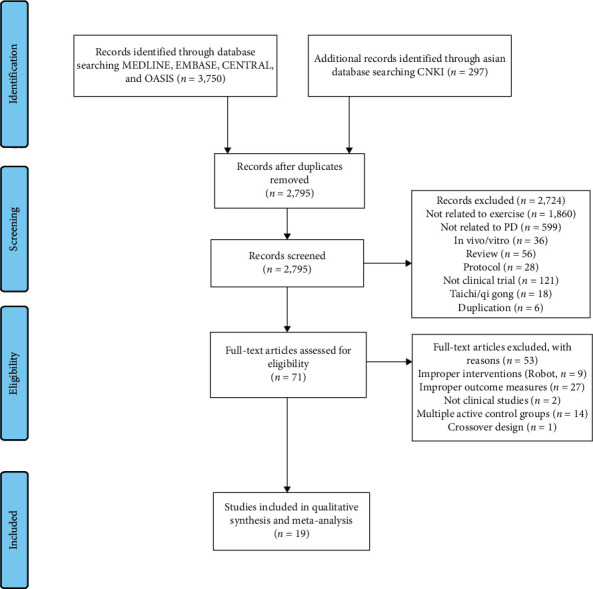
PRISMA flow chart of the study selection and identification process [22].

A total of 4,047 studies were retrieved by electronic search. After eliminating duplicates, the 2,795 studies left were screened by abstract. Among them, 71 studies were selected for eligibility assessment. After reviewing the full texts, 18 studies (1,144 patients) were finally selected for the meta-analysis. Fifty-three studies were excluded due to the following reasons: improper interventions such as robot therapies (*n* = 9), inappropriate outcome measures (*n* = 27), ineligible study design (*n* = 3), and inappropriate control group which contained more than 2 active control groups (*n* = 14) (Figure 1, Table 2).

Among the 18 final studies, 12 were reported in English and 6 were reported in Chinese (Table 3).

Disease duration and symptom severity (Hoehn and Yahr scale) showed large variations among the included literature. All studies [23–40] used anti-Parkinsonian medications as usual therapies regardless of the intervention and performed the outcome measurements in one period.

The intervention period ranged from a minimum of one month [34] to a maximum of 14 months [27]. The frequency of the intervention performed was different for each study. The types of interventions were as follows: walking exercise (*n* = 3) [23–25], strength exercise (*n* = 4) [26–29], balancing exercise (*n* = 2) [30, 31], aerobic exercise (*n* = 4) [32–35], and complex exercise (*n* = 5) [36–40]. In all the studies [23–40], participants maintained their usual daily activities outside of the trial, including anti-Parkinsonian medication. The exercise duration in each session varied from 10 minutes [34] to 3 hours [37]. Most studies were performed 30–60 minutes (*n* = 13) regardless of the type of exercise [23–25, 28–33, 35, 36, 38, 39].

The frequency of the exercises also varied. Most literature (*n* = 4) [24, 25, 30, 31] conducted exercise three times a week. Three studies carried out exercise once a week [26, 33, 38], two studies twice a week [32, 35], other three studies five times a week [28, 37, 39], two studies every day [27, 36], one study four times a week [23], and another study five or six times a week [29]. However, there was one study that did not report exercise frequency [40].

There were two types of comparisons. (1) exercise + conventional medications vs. conventional medications only (nonexercise) (*n* = 15) [23, 27–40] and (2) exercise + conventional medications vs. regular activity + conventional medications (*n* = 3) [24–26]. The types of regular activities varied, such as social interaction, life skill program, and conventional physical therapy. These were performed in the same manner as in the exercise treatment group.

Regarding the evaluation scale, UPDRS part III was the most common evaluation scale in 9 studies [23, 24, 26–28, 31, 32, 35, 37]. For balance evaluation, the BBS was used in a total of 6 articles [28, 29, 33, 36, 38, 39], and the TUGT was used in 5 articles [26, 33, 35, 38, 39]. Gait velocity and the 6-minute walk test (6 MWT) were the evaluation scale for walking ability. The gait velocity was evaluated by two methods: the preferred speed (m/s) and the fast speed (m/s). Four articles [24–26, 30] used the preferred speed (m/s), and one article [34] used the fast speed (m/s). Two articles [23, 25] used the 6 MWT as the evaluation scale.

### 3.2. Risk of Bias within Studies

**Figure 2:**
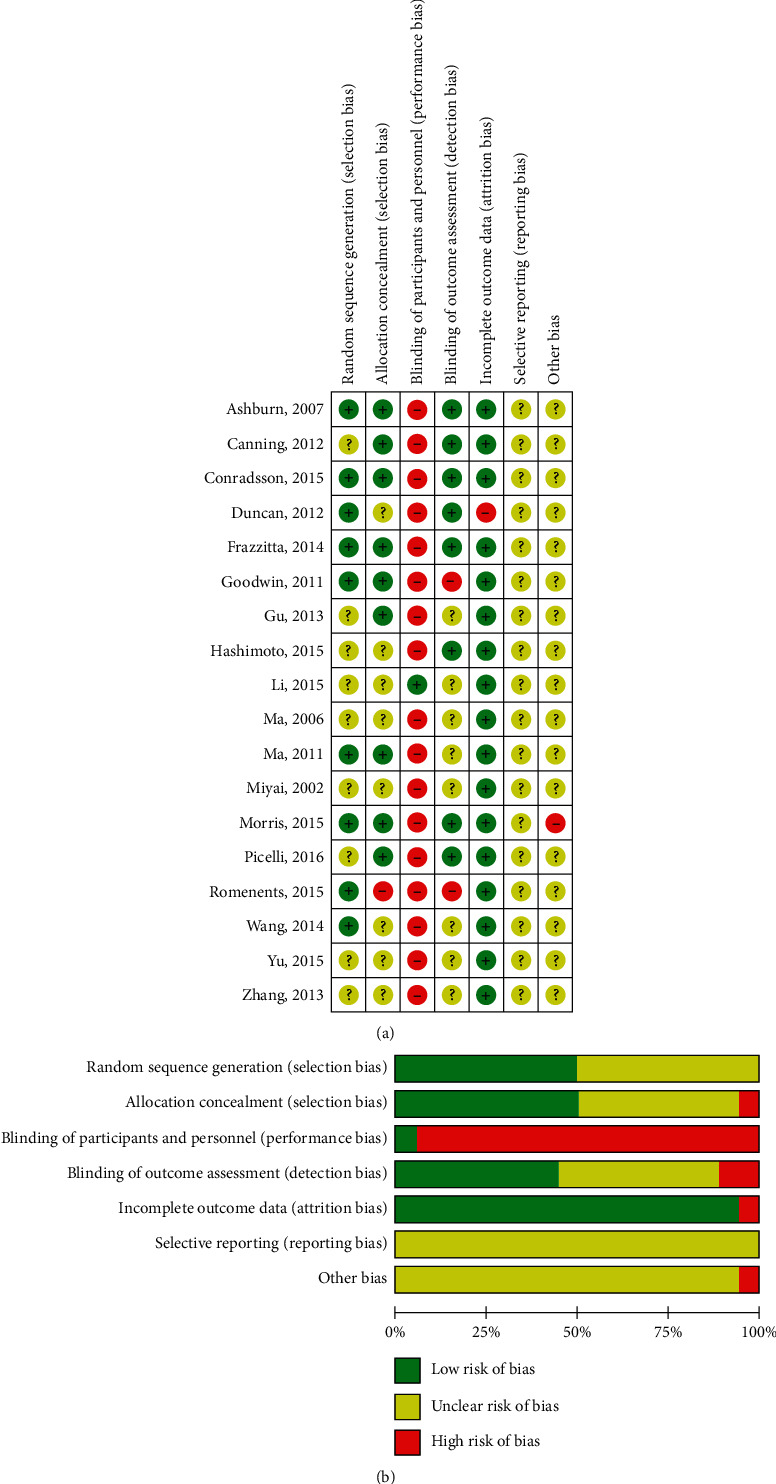
(a) The graph of risk of bias; (b) the summary of risk of bias: “+” = low risk of bias, “−” = high risk of bias, and “?” = unclear risk of bias.

In most studies, the risk of bias was not high. Among the risk of bias domains, blinding of the participants and personnel and selective reporting revealed methodological concerns. Nine articles [23–25, 27, 29, 31, 33, 39, 40] were classified as ‘unclear risk of bias' in the random sequence generation because there was no specific description of the randomization method. Eight studies [24, 27–29, 32, 33, 39, 40] were classified as “unclear risk of bias” in the allocation concealment. Another study [35] that did not conceal the assignment order was classified as “high risk of bias.” Most studies were classified as “high risk of bias” in the blinding of participants (performance bias) [23–26, 28–40]. In the incomplete outcome data (attrition bias), one study [32] was evaluated as “high risk of bias” and all the remaining studies were evaluated as “low risk of bias”. In the selective reporting (reporting bias), one study [26] was rated as “high risk of bias” and the rest of the studies were evaluated as “unclear risk of bias.” A summary of the risk of bias is shown in Figure 2.

### 3.3. Total Unified Parkinson's Disease Rating Scale (UPDRS) Scores

Three studies [24, 33, 40] used the total UPDRS scores, five articles [24, 26, 27, 30, 31] used UPDRS II, and 9 studies [23, 24, 26–28, 31, 32, 35, 37] used UPDRS III.

**Figure 3:**
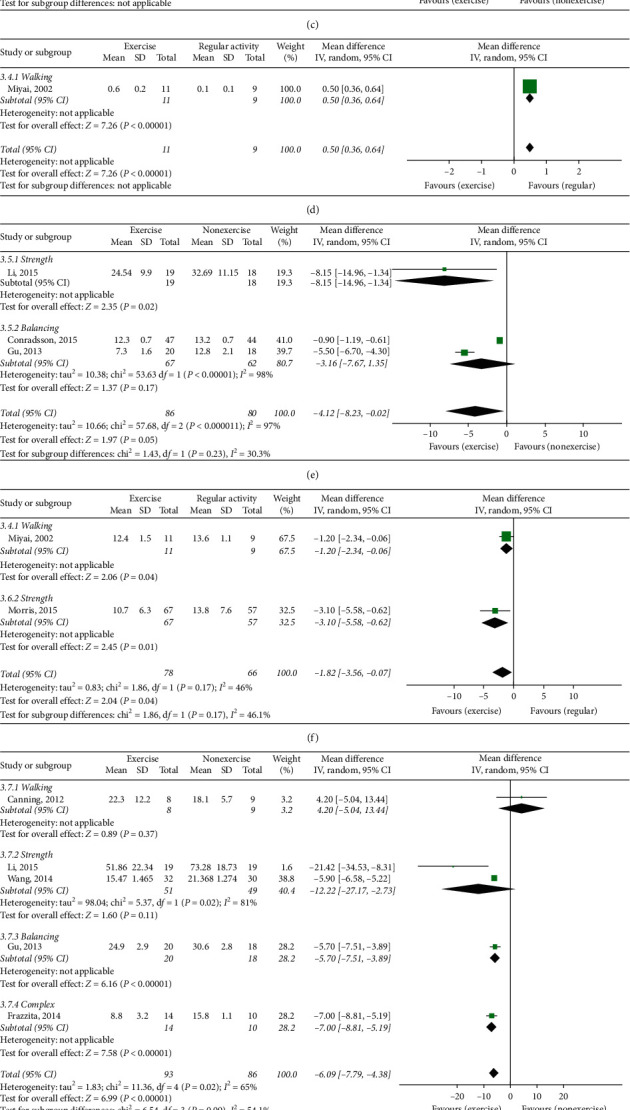
(a) Total UPDRS score, exercise therapy group versus nonexercise group. (b) Total UPDRS score, exercise therapy group versus regular activity group. (c) UPDRS I exercise therapy group versus nonexercise group. (d) UPDRS I exercise therapy group versus regular activity group. (e) UPDRS II, exercise therapy group versus nonexercise group. (f) UPDRS II, exercise therapy group versus regular activity group. (g) UPDRS III, exercise therapy group versus nonexercise group. (h) MDS-UPDRS III, exercise therapy group versus nonexercise group. (i) UPDRS III, exercise therapy group versus regular activity group.

Two studies [33, 40] compared the total UPDRS score in the exercise therapy group (ET) to that of the nonexercise group (NE). ET showed a significant effect on the total UPDRS score (MD −16.84, 95% CI (−22.52, −11.16)). In the subgroup analysis based on the type of exercise, there were significant results in the ET (aerobic exercise [33]: MD −14.20, 95% CI (−22.66, −5.74); complex exercise [40]: MD −19.00 and 95% CI (−26.66, −11.44)) (Figure 3(a)).

One study [24] compared the exercise therapy group (ET, walking exercise) with the regular activity group (RA). ET showed a significant effect in the total UPDRS score (MD −2.90, 95% CI (−5.44, −0.36)) [24] (Figure 3(b)).

### 3.4. UPDRS I Scores

Two studies [24, 31] used UPDRS I. There was a study [31] evaluating the effect of balancing exercise and it compared the UPDRS I subscore in ET and NE. ET showed a positive effect in the total UPDRS I (MD −0.10, 95% CI (−0.26, 0.06)) (Figure 3(c)). There was another study [24] that used walking exercise and compared the UPDRS I subscore between ET and RA. In this study, ET did not show a positive effect in the UPDRS I (MD 0.50, 95% CI (0.36, 0.64)) (Figure 3(d)).

### 3.5. UPDRS II Scores

Three studies (including 166 patients) [27, 30, 31] evaluated the UPDRS II score in ET and NE. ET showed a significant effect on the UPDRS II score (MD −4.12 and 95% CI (−8.23, −0.02)). In the subgroup analysis based on the type of exercise, there was also a significant effect in strength exercise (MD −8.15, 95% CI (−14.96, −1.34)) [27] (Figure 3(e)).

Additional two studies [24, 26] compared ET with RA. ET showed a significant effect on the UPDRS II score (MD −1.82, 95% CI (−3.56, −0.07)) (Figure 3(f)).

### 3.6. UPDRS III Scores

Five studies (including 179 patients) [23, 27, 28, 31, 37] compared UPDRS III scores in ET and NE. ET showed a significant effect on the UPDRS III score (MD −6.09 and 95% CI (−7.79, −4.38)). In the subgroup analysis, there were inconsistencies depending on the exercise type. Walking [23] and strength [27, 28] exercise did not show a positive effect, and balancing [31] and complex exercises [37] showed significant effects (Figure 3(g)).

Eighty-five Parkinson's disease patients in two studies [32, 35] divided into ET and NE were evaluated by MDS-UPDRS III. ET showed a significant effect in the UPDRS III score (MD −11.69 and 95% CI (−16.96, −6.42)) (Figure 3(h)).

On the other hand, two studies (144 patients) [24, 26] compared ET with RA. ET showed a significant effect in the UPDRS III score (MD −2.53, 95% CI (−3.75, −1.31)) (Figure 3(i)).

### 3.7. Berg Balance Scale (BBS)

**Figure 4:**
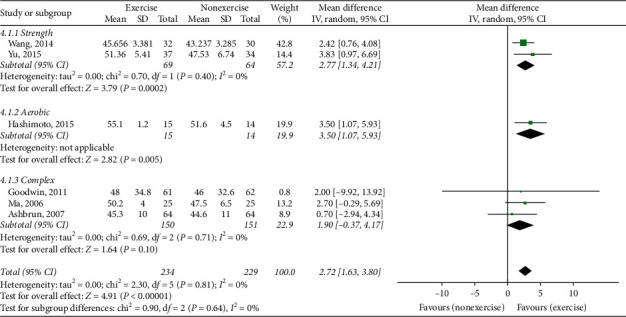
Berg Balance Scale, exercise therapy group versus nonexercise group.

Six studies (463 patients) [28, 29, 33, 36, 38, 39] compared ET with NE. ET showed a significant effect on the BBS scores (MD 2.72 and 95% CI (1.63, 3.80)). In the subgroup analysis, strength [28, 29] and aerobic exercise [33]showed significant effects on BBS scores (Figure 4).

### 3.8. Preferred Walking Speed

**Figure 5:**
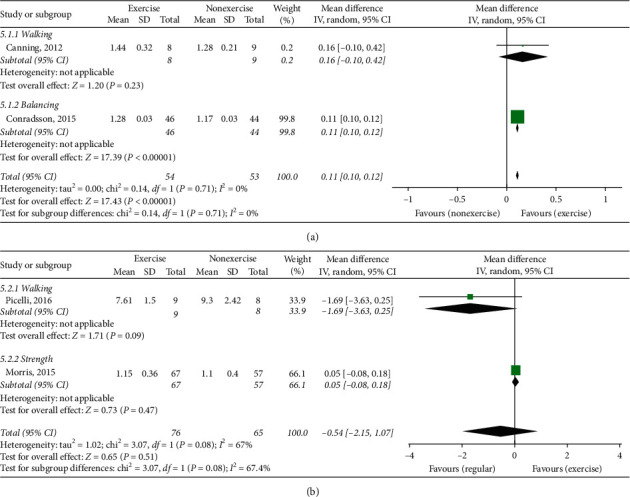
(a) Preferred walking speed, exercise therapy group versus nonexercise group. (b) Preferred walking speed, exercise therapy group versus regular activity group.

There were 4 studies [24–26, 30] that used preferred walking speed to evaluate the gait function. Among them, two studies (107 patients) [23, 30] compared ET with NE. There was a significant difference in the preferred walking speed between the two groups (MD 0.11, 95% CI (0.10, 0.12)). In the subgroup analysis, only balancing exercise [30] revealed a significant effect (Figure 5(a)).

Two other studies (141 patients) [25, 26] compared ET with RA. No significant differences in the preferred walking speed were shown between the two groups (MD -0.54, 95% Cl (−2.15, 1.07)) (Figure 5(b)).

### 3.9. Fast Walking Speed

**Figure 6:**
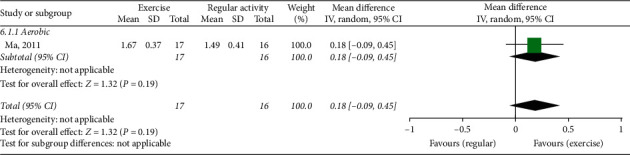
Fast walking speed, exercise therapy group versus nonexercise group (placebo exercise).

One study (including 33 patients) [34] compared ET with NE (placebo exercise). No significant differences in fast walking speed were shown between the groups (MD 0.18, 95% CI (−0.09, 0.45)) (Figure 6).

### 3.10. Timed Up and Go Test (TUGT)

**Figure 7:**
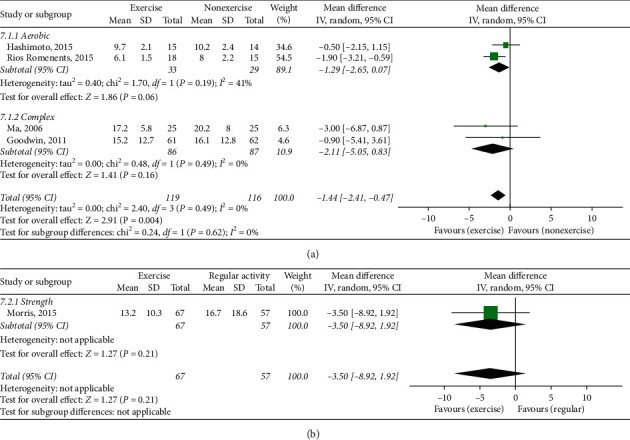
(a) Timed Up and Go Test, exercise therapy group versus nonexercise group. (b) Timed Up and Go Test, exercise therapy group versus regular activity group.

Five studies used the Time Up and Go Test (TUGT) for evaluating gait function. Among them, four studies (235 patients) [33, 35, 38, 39] compared between ET and NE. ET showed a significant difference between groups (MD −1.44, 95% CI (−2.41, −0.47)) (Figure 7(a)).

Another study (including 124 patients) [26] compared ET with RA. No significant difference in the TUGT was shown between the groups (MD −3.50, 95% CI (−8.92, 1.92)) (Figure 7(b)).

### 3.11. Six-Minute Walk Test (6 MWT)

**Figure 8:**
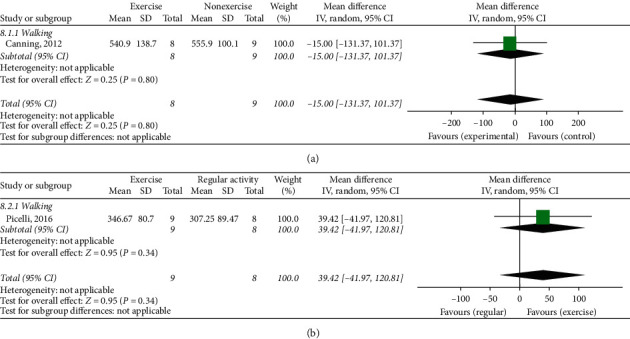
(a) Six-minute walk test, exercise therapy group versus nonexercise group. (b) Six-minute walk test, exercise therapy group versus regular activity group.

Two studies used the 6-minute walk test (6 MWT) to evaluate gait function. One study (including 17 patients) [23] compared ET with NE. ET did not show a significant effect in the 6 MWT (Figure 8(a)). Another study (including 17 patients) [25] compared ET with RA. In this analysis, ET did not show a significant effect in the 6 MWT (Figure 8(b)).

### 3.12. Safety

Among the 18 articles, only seven articles [23, 25, 26, 30, 34, 35, 38] investigated adverse effects due to the interventions. Of these, there were no adverse events reported in four studies [23, 25, 26, 38]. Falls (*n* = 14) and fatigue (*n* = 2) were reported as adverse effects in three studies [30, 34, 35], and respiratory infection (*n* = 1) which was not related to the intervention was reported in one study [35].

## 4. Discussion

The results of this systematic review and meta-analysis show that ET improved motor and nonmotor symptoms in PD compared with NE or RA. ET showed a significant improvement in UPDRS (total, II, III, and MDS-UPDRS III) scores, BBS, preferred walking speed, and TUGT compared to NE and UPDRS (total, II, and III) compared to RA.

Previously, several meta-analyses had been reported which evaluated exercise interventions in patients with PD [10, 41–45]. The differences between the previous studies and the present study are as follows. First, in this study, various interventions and outcomes were investigated. Most of the previous studies were limited in the specific type of exercise therapy [10, 43, 44] or the specific type of outcome measure [45]. Another study only showed the characteristics, intervention delivery, retention rates, adherence, and adverse events of exercise therapies [42]. However, there was no report about improvement of PD symptoms. In this study, we aimed to comprehensively evaluate the effects of various types of exercise therapies on PD and to evaluate the effects of each type of exercise therapy through subgroup analysis. Therefore, we tried to classify exercise interventions into 5 groups according to four types of exercise therapy and to provide a summary effect estimate of the individual exercise types. At the same time, we extracted various outcome measures such as total UPDRS and UPDRS part I, II, III, and IV, BBS, TUGT, and gait velocity (preferred speed, fast speed, 6MWT). Therefore, the relationship between the improvement of motor or nonmotor symptoms of PD and exercise therapies could be evaluated. The degree of improvements of symptoms according to the type of exercise could be known. Based on these results, it is possible to apply it to clinical care. Second, this study analyzed a larger number of literature, being an update of existing studies. There were 495 PD patients included in 14 articles in the previous studies of various exercise interventions as classified in this study [41]. In this study, we included 1,144 PD patients in 18 studies. Therefore, it could be assessed that the reliability of our study was increased. Finally, we tried to reduce the heterogeneity and to obtain accurate results by dividing the results into two groups according to whether regular activity was performed in the control group or not. In addition to the nonexercise group (NE), we evaluated the regular activity (RA) group to confirm that the results are different. We found that even simple activities could also help to improve symptoms of PD.

In this study, exercise therapies have been shown to be effective in improving the overall symptoms of PD, the activities of daily life (ADLs) related to motor function, overall motor symptoms, balance, and gait disturbance. The effects of each exercise type are as follows: walking exercises showed significant effects on ADLs related to motor function and motor symptoms compared with RA; strength and flexibility exercises revealed significant effects on ADLs related to motor function (compared with NE and RA) and balance (compared with NE); balancing exercise has significant effects on motor symptoms and gait disturbance (compared with NE); aerobic exercise showed significant effects on motor symptoms and balance (compared with NE); and complex exercise revealed a significant effect on motor symptoms. As mentioned above, depending on the type of exercise, we could see the difference in the degree to which PD symptoms were improved. There is a high heterogeneity of the resulting values because of differences in the duration and method of exercise therapy for each study included in the meta-analysis. Nonetheless, in clinical applications, clinicians will be able to make appropriate and flexible use of the results of this study, depending on the circumstances and experience (Table 4). Adverse events such as falling and fatigue have been reported. Among them, the most common was falling in two articles [30, 35]. Falling was observed only in balancing and aerobic exercise, 13 out of 14 occurred during balancing exercise. According to a review article [46], postural instability is known to be observed in 16% of PD patients. Postural instability gradually deteriorates as the disease progresses, which is the main cause of falling [47]. If there are patients with severe postural instability, the balancing exercise should be considered carefully. Other types of exercise (besides the balancing exercise) did not show any severe adverse effects other than fatigue. Therefore, they could be applied to PD patients relatively more safely.

The limitations of this study are as follows: first, there is a bias in the literature included in the aspects of qualitative research methodology. Selection bias may exist because random sequence generation or allocation concealment were not specifically addressed [23–25, 27–29, 31–33, 39, 40] or one study [35] was evaluated as “high risk of bias” because of the absence of blinding of participants (performance bias). Only one study [27] conducted blinding of participants. Also, in the blinding of outcome assessment, two studies [35, 38] were evaluated as “high risk of bias.” Therefore, selection bias and detection bias may have influenced the result of this study. Second, heterogeneity is high. This is thought to be because there was a huge difference in the quality of the studies, the patients participating in the study, and the exercise treatment and regular activity in each literature. Third, the sample sizes of the literatures included in this study are still small. This study was divided into two groups according to whether regular activity was performed in the control group and subgrouped by the types of intervention (walking, strength, balancing, aerobic, and complex exercises). Therefore, since a total of 18 articles were divided, the number of articles included in each evaluation index was quite small. This might have led to the lower test effectiveness. Therefore, further follow-up research with additional exercise therapy intervention clinical papers should be conducted.

## 5. Conclusions

Exercise therapies might promote improvements in the motor symptoms and related ADLs of PD. Overall, exercise therapies showed significant effects on motor function of PD patients compared to NE group or RA group. In contrast, exercises did not show a statistically significant effect on nonmotor symptoms compared to the NE group or RA group. These results suggest that exercise therapy is more effective for motor symptoms of PD patients rather than nonmotor symptoms. However, due to the small number of randomized controlled trials and methodological limitations, we are unable to draw concrete conclusions. Therefore, further studies with better designs will be needed.

## Figures and Tables

**Table 1:** Search strategy.

MEDLINE	
Search	Query

#1	Search “Parkinson Disease”[Mesh] OR “Parkinsonian Disorders”[Mesh]
#2	Search “Parkinson Disease”[tiab] OR “Parkinsonian Disorders”[tiab]
#3	Search Parkinson∗[tiab]
#4	Search ((Parkinson∗[tiab]) OR (“Parkinson Disease”[tiab] OR “Parkinsonian Disorders”[tiab])) OR (“Parkinson Disease”[Mesh] OR “Parkinsonian Disorders”[Mesh])
#5	Search (((((((((“Exercise Therapy”[Mesh]) OR “Physical therapy Modalities”[Mesh]) OR “Exercise Test”[Mesh]) OR “Exercise movement Techniques”[Mesh]) OR “occupational Therapy”[Mesh]) OR “Physical Fitness”[Mesh]) OR “Movement”[Mesh]) OR “physical Stimulation”[Mesh]) OR “Physical education and Training”[Mesh]) OR “physical and rehabilitation Medicine”[Mesh]
#6	Search (((((((((“Exercise Therapy”[tiab]) OR “Physical therapy Modalities”[tiab]) OR “exercise Test”[tiab]) OR “Exercise Movement Techniques”[tiab]) OR “occupational Therapy”[tiab]) OR “physical Fitness”[tiab]) OR “Movement”[tiab]) OR “physical Stimulation”[tiab]) OR “physical education and Training”[tiab]) OR “physical and rehabilitation Medicine”[tiab]
#7	Search Treadmil$[tiab] OR excercise$[tiab]
#8	Search (((Treadmil$[tiab] OR excercise$[tiab])) OR ((((((((((“exercise Therap”[tiab]) OR “physical therapy Modalities”[tiab]) OR “exercise Test”[tiab]) OR “exercise movement Techniques”[tiab]) OR “occupational Therapy”[tiab]) OR “physical Fitness”[tiab]) OR “Movement”[tiab]) OR “physical Stimulation”[tiab]) OR “physical education and Training”[tiab]) OR “physical and rehabilitation Medicine”[tiab])) OR ((((((((((“exercise Therapy”[Mesh]) OR “physical therapy Modalities”[Mesh]) OR “exercise Test”[Mesh]) OR “exercise movement Techniques”[Mesh]) OR “occupational Therapy”[Mesh]) OR “physical Fitness”[Mesh]) OR “Movement”[Mesh]) OR “physical Stimulation”[Mesh]) OR “physical education and Training”[Mesh]) OR “physical and rehabilitation Medicine”[Mesh])
#9	Search (((((Treadmil$[tiab] OR excercise$[tiab])) OR ((((((((((“exercise Therap”[tiab]) OR “physical therapy Modalities”[tiab]) OR “exercise Test”[tiab]) OR “exercise movement Techniques”[tiab]) OR “occupational Therapy”[tiab]) OR “physical Fitness”[tiab]) OR “Movement”[tiab]) OR “physical Stimulation”[tiab]) OR “physical education and Training”[tiab]) OR “physical and rehabilitation Medicine”[tiab])) OR ((((((((((“Exercise Therapy”[Mesh]) OR “physical therapy Modalities”[Mesh]) OR “Exercise Test”[Mesh]) OR “Exercise movement Techniques”[Mesh]) OR “occupational Therapy”[Mesh]) OR “physical Fitness”[Mesh]) OR “Movement”[Mesh]) OR “physical Stimulation”[Mesh]) OR “physical education and Training”[Mesh]) OR “physical and rehabilitation Medicine”[Mesh]))) AND (((Parkinson∗[tiab]) OR (“Parkinson Disease”[tiab] OR “parkinsonian Disorders”[tiab])) OR (“Parkinson Disease”[Mesh] OR “parkinsonian Disorders”[Mesh]))
#10	Search (((((Treadmil$[tiab] OR excercise$[tiab])) OR ((((((((((“exercise Therapy”[tiab]) OR “physical therapy Modalities”[tiab]) OR “exercise Test”[tiab]) OR “exercise movement Techniques”[tiab]) OR “occupational Therapy”[tiab]) OR “physical Fitness”[tiab]) OR “Movement”[tiab]) OR “physical Stimulation”[tiab]) OR “physical education and Training”[tiab]) OR “physical and rehabilitation Medicine”[tiab])) OR ((((((((((“exercise Therapy”[Mesh]) OR “physical therapy Modalities”[Mesh]) OR “Exercise Test”[Mesh]) OR “exercise movement Techniques”[Mesh]) OR “occupational Therapy”[Mesh]) OR “physical Fitness [Mesh]) OR “Movement”[Mesh]) OR “physical Stimulatio”[Mesh]) OR “physical education and Training”[Mesh]) OR “physical and rehabilitation Medicine”[Mesh]))) AND (((Parkinson∗[tiab]) OR (“Parkinson Disease”[tiab] OR “parkinsonian Disorders”[tiab])) OR (“Parkinson Disease”[Mesh] OR “parkinsonian Disorders”[Mesh])) filters: Clinical trial

CENTRAL	
ID	Search
#1	MeSH descriptor: [Parkinson disease] explode all trees
#2	Parkinson∗:ti,ab, kw (word variations have been searched)
#3	MeSH descriptor: [Physical therapy modalities] explode all trees
#4	MeSH descriptor: [Exercise movement techniques] explode all trees
#5	MeSH descriptor: [Movement] explode all trees
#6	MeSH descriptor: [Physical fitness] explode all trees
#7	MeSH descriptor: [Occupational therapy] explode all trees
#8	MeSH descriptor: [Physical endurance] explode all trees
#9	MeSH descriptor: [Physical stimulation] explode all trees
#10	MeSH descriptor: [Physical education and training] explode all trees
#11	MeSH descriptor: [Physical and rehabilitation medicine] explode all trees
#12	MeSH descriptor: [Exercise therapy] explode all trees
#13	MeSH descriptor: [Physical therapy modalities] explode all trees
#14	“Physical therapy” or “exercise movement techniques” or “occupational therapy” or movement or “physical fitness”: ti, ab, kw (word variations have been searched)
#15	“Physical endurance” or “physical stimulation” or “physical education” or “physical: ti, ab, kw (word variations have been searched)
#16	Walking∗ or Treadmil$∗: ti,ab, kw (word variations have been searched)
#17	#1 or #2
#18	#3 or #4 or #5 or #6 or #7 or #8 or #9 or #10 or #11 or #12 or #13 or #14 or #15 or #16
#19	#17 and #18

EMBASE	
No.	Query
#1	“Parkinson disease”/exp
#2	Parkinson∗:ab,ti
#3	“Parkinson disease”/exp OR Parkinson∗:ab,ti
#4	“physiotherapy”/exp OR “kinesiotherapy”/exp OR “occupational therapy”/exp OR “body movement”/exp OR “fitness”/exp OR “endurance”/exp OR “stimulation”/exp OR “physical education”/exp OR “physical medicine”/exp OR “exercise”/exp
#5	“physiotherapy”:ab, ti OR “kinesiotherapy”:ab, ti OR “occupational therapy”:ab, ti OR “body movement”:ab, ti OR “fitness”:ab, ti OR “endurance”:ab, ti OR “stimulation”:ab, ti OR “physical education”:ab, ti OR “physical medicine”:ab, ti OR “exercise”:ab,ti
#6	walking∗:ab, ti OR treadmil∗:ab,ti
#7	(“physiotherapy”/exp OR “kinesiotherapy”/exp OR “occupational therapy”/exp OR “body movement”/exp OR “fitness”/exp OR “endurance”/exp OR “stimulation”/exp OR “physical education”/exp OR “physical medicine”/exp OR “exercise”/exp) OR (“physiotherapy”:ab, ti OR “kinesiotherapy”:ab, ti OR “occupational therapy”:ab, ti OR “body movement”:ab, ti OR “fitness”:ab, ti OR “enduranc”:ab, ti OR “stimulation”:ab, ti OR “physical education”':ab, ti OR “physical medicine”:ab, ti OR “exercise”:ab,ti) OR (walking∗:ab, ti OR treadmil∗:ab,ti)
#8	(“Parkinson disease”/exp OR Parkinson∗:ab,ti) AND ((“physiotherapy”/exp OR “kinesiotherapy”/exp OR “occupational therapy”/exp OR “body movement”/exp OR “fitness”/exp OR “endurance”/exp OR “stimulation”/exp OR “physical education”/exp OR “physical medicine”/exp OR “exercis”/exp) OR (“physiotherapy”:ab, ti OR “kinesiotherapy”:ab, ti OR “occupational therapy”:ab, ti OR “body movement”:ab, ti OR “fitness :ab, ti OR “endurance”:ab, ti OR “stimulation”:ab, ti OR “physical education”:ab, ti OR “physical medicine”:ab, ti OR “exercise”:ab,ti) OR (walking∗:ab, ti OR treadmil∗:ab,ti))
#9	(“Parkinson disease”/exp OR Parkinson∗:ab,ti) AND ((“physiotherapy”/exp OR “kinesiotherapy”/exp OR “occupational therapy”/exp OR “body movement”/exp OR “fitness”/exp OR “endurance”/exp OR “stimulation”/exp OR “physical education”/exp OR “physical medicine”/exp OR “exercise”/exp) OR (“physiotherapy”:ab, ti OR “kinesiotherapy”:ab, ti OR “occupational therapy”:ab, ti OR “body movement”:ab, ti OR “fitness”:ab, ti OR “endurance”:ab, ti OR “stimulation”:ab, ti OR “physical education”:ab, ti OR “physical medicine”:ab, ti OR “exercise”:ab,ti) OR (walking∗:ab, ti OR treadmil∗:ab,ti)) AND [humans]/lim
#10	(“Parkinson disease”/exp OR Parkinson∗:ab,ti) AND ((“physiotherapy”/exp OR “kinesiotherapy”/exp OR “occupational therapy”/exp OR “body movement”/exp OR “fitness”/exp OR “endurance”/exp OR “stimulation”/exp OR “physical education”/exp OR “physical medicine”/exp OR “exercise”/exp) OR (“physiotherapy”:ab, ti OR “kinesiotherapy”:ab, ti OR “occupational therapy”:ab, ti OR “body movement”:ab, ti OR “fitness”:ab, ti OR “endurance”:ab, ti OR “stimulation”:ab, ti OR “physical education”:ab, ti OR “physical medicine”:ab, ti OR “exercise”:ab,ti) OR (walking∗:ab, ti OR treadmil∗:ab,ti)) AND [humans]/lim AND ([controlled clinical trial]/lim OR [randomized controlled trial]/lim)
#11	(“Parkinson disease”/exp OR Parkinson∗:ab,ti) AND ((“physiotherapy”/exp OR “kinesiotherapy”/exp OR “occupational therapy”/exp OR “body movement”/exp OR “fitness”/exp OR “endurance”/exp OR “stimulation”/exp OR “physical educatio”/exp OR “physical medicine”/exp OR “exercise”/exp) OR (“physiotherapy”:ab, ti OR “kinesiotherapy”:ab, ti OR “occupational therapy”:ab, ti OR “body movement”:ab, ti OR “fitness”:ab, ti OR “endurance”:ab, ti OR “stimulation”:ab, ti OR “physical education”:ab, ti OR “physical medicine”:ab, ti OR “exercise”:ab,ti) OR (walking∗:ab, ti OR treadmil∗:ab,ti)) AND [humans]/lim AND ([controlled clinical trial]/lim OR [randomized controlled trial]/lim) AND ([cochrane review]/lim OR [systematic review]/lim OR [meta-analysis]/lim)

OASIS	
파킨슨 and 운동	

CNKI	
#1	帕金森病
#2	帕金森氏病
#3	震颤麻痹
#4	颤病
#5	颤证
#6	颤震
#7	颤拘病
#8	振掉
#9	拘病
#10	Parkinson disease
#11	Or/#1-#10
#12	功能锻炼
#13	运动训练
#14	锻炼
#15	康复运动训练
#16	OR/#12-#15
#17	#11 AND #17

**Table 2:** Dropout reasons of trials.

First author and year	Dropout reason
Frazzitta, 2015	Improper intervention (robot)
Picelli, 2012(A)	Improper intervention (robot)
Picelli, 2012(B)	Improper intervention (robot)
Picelli, 2013	Improper intervention (robot)
Picelli, 2015	Improper intervention (robot)
Pompeu, 2012	Improper intervention (robot)
Shih, 2016	Improper intervention (robot)
Yang, 2016	Improper intervention (robot)
Heuvel, 2014	Improper intervention (robot)
Fietzek, 2014	Crossover design
Schenkman, 2012	Improper interventions (not exercise)
Rong, 2016	Improper outcome measures
Ma, 2006	Improper outcome measures
Liú, 2016	Improper outcome measures
Allen, 2010	Improper outcome measures
Canning, 2015	Improper outcome measures
DiFrancisco-Donoghue, 2012	Improper outcome measures
Fok, 2012	Improper outcome measures
Kurtais, 2008	Improper outcome measures
Lee, 2011	Improper outcome measures
Mak, 2008	Improper outcome measures
Martin, 2015	Improper outcome measures
Mateos-Toset, 2016	Improper outcome measures
McGinley, 2012	Improper outcome measures
Nimwegen, 2013	Improper outcome measures
Olmo, 2006	Improper outcome measures
Platz, 1998	Improper outcome measures
Prodoehl, 2015	Improper outcome measures
Reuter, 2011	Improper outcome measures
Rios Romenets, 2013	Improper outcome measures
Rose, 2013	Improper outcome measures
Schenkman, 1998	Improper outcome measures
Shen, 2015	Improper outcome measures
Stack, 2012	Improper outcome measures
Sturkenboom, 2014	Improper outcome measures
Teixeira-Machado, 2015	Improper outcome measures
Wong-Yu, 2015(A)	Improper outcome measures
Yang, 2010	Improper outcome measures
Landers, 2016	Many active control groups
Shulman, 2013	Many active control groups
Kolk, 2015	Not a clinical study (protocol)
Nimwegen, 2010	Not a clinical study (protocol)
Bello, 2013	Many active control groups
Fernandez del Olmo, 2014	Many active control groups
Harro, 2014	Many active control groups
Sale, 2013	Many active control groups
Uc, 2014	Many active control groups
Paul, 2014	Many active control groups
Volpe, 2014	Many active control groups
Wong-Yu, 2015	Many active control groups
Dibble, 2015	Many active control groups
Schlenstedt, 2015	Many active control groups
Shen, 2014	Many active control groups

**Table 3:** Summary of the included studies.

Study. First author, year	Participants Sample size (E/C) PD duration (E/C) (range or mean ± SD) H&Y (range or mean ± SD)	Intervention	Regimen	Control	Outcome measures	Adverse events
1. Walking exercise						
Canning, 2012 [23]	20 (10/10) 6.1 ± 4.0/5.2 ± 4.1 N/A	W: comfortable gait speed on treadmill (hold on to the handrails)	30–40 min, 4 times/wk for 6 wks	Usual care	6 MWT PS UPDRS III	No adverse events
Miyai, 2002 [24]	20 (11/9) 4.1 ± 0.8/4.5 ± 0.7 2.9 ± 0.1/2.8 ± 0.1	W: treadmill training (body weight supported) 10 min walking with 20% of BWS⟶10 min walking with 10% of BWS⟶10 min walking with 0% of BWS⟶15 min rest. Treadmill speed was initiated at 0.5 km/h and increased to 3.0 km/h by increments of 0.5 km/h as tolerated	45 min, 3 times/wk for 1 Mon	Conventional physical therapy 45 min, 3 times/wk for 1 Mon	Total UPDRS UPDRS I UPDRS II UPDRS III	N/A
Picelli, 2016 [25]	17 (9/8) 11.2 ± 5.6/10.8 ± 4.1 3 (all participants)	W: treadmill training without body weight support. Each training session comprised three parts with a 5 min rest after each session. The speed of 1.0 km/h for 10 min ⟶ 1.5 km/h for 10 min ⟶ 2.0 km/h for 10 min	45 min, 3 times/wk for 4 wks	Regular social interactions 45 min, 3 times/wk for 4 wks	6 MWT PS	No adverse events

2. Strength and flexibility exercise						
Morris, 2015 [26]	203 (70/68/65) 6.7 ± 5.6 (all participants) 1–4 (all participants)	S: progressive resistance strength training. Exercises were progressed by increasing: repetitions to a maximum of 15, sets to a maximum of 3, or weights by 2% of the person's body weight	2 hr, 1 time/wk for 8 wks	C1 : movement strategy training (active control) 2 hr, 1 time/wk for 8 wks C2 : life skills program 2 hr, 1 time/wk for 8 wks	UPDRS II UPDRS III PS TUGT	No serious adverse events
Li, 2015 [27]	38 (19/19) 8.71 ± 6.23/7.21 ± 5.53 2–3 (all participants)	S: physical exercises trunk stretching, section stretching, lower extremities stretching, hold knee in one's arms, upper extremities stretching, walking, turn waist, punching, running, athetotic gait exercise, and kinematic contact exercise	120 min, 7 times/wk for 14 Mons	Usual care	UPDRS II UPDRS III	N/A
Wang, 2014 [28]	64 (32/30) N/A N/A	S: physical exercises divided into 8 methods	30–45 min, 5 times/wk for 8 wks twice a day	Usual care	UPDRS III BBS	N/A
Yu, 2015 [29]	71 (37/34) 3.9 ± 1.4/4.1 ± 1.3 3 (all participants)	S: core muscular strengthening exercise. Trunk anteflexion, extension, lateroflexion, and rotation. Abdominal muscles exercise, hold knee in one's arms in the supine position, straight leg raising in the supine position, and Fowler's position change. Lumbodorsal strengthening exercise, gluteus strengthening exercise, and straight leg raising in the pronation position	30 min, 2 times/d, 5-6 times/wk for 3 Mons	Usual care	BBS	N/A

3. Balancing exercise						
Conradsson, 2015 [30]	91 (47/44) 6.0 ± 5.1/5.6 ± 5.0 2-3 (all participants)	B: plus cognitive and/or motor tasks 1. Sensory integration (walking tasks on varying surfaces with or without visual constraints) 2. Anticipatory postural adjustments (voluntary arm/leg/trunk movements, postural transitions, and multidirectional stepping, and emphasizing movement velocity and amplitude) 3. Motor agility (interlimb coordination under varying gait conditions and quick shifts of movement characteristic during predictable and unpredictable conditions) 4. Stability limits (controlled leaning tasks performed while standing with varying bases of support, stimulating weight shifts in multiple directions)	60 min, 3 times/wk for 10 wks	Usual care	UPDRS II PS	Total 13 adverse events (fallings during training)
Gu, 2013 [31]	38 (20/18) 5.8 ± 1.9/6.2 ± 2.1 1–3 (all participants)	B: 1. Standing with gathering legs, standing after tandem gait, stand on one foot, standing for a long time, and standing with eyes closed. 2. Standing holding a thing. 3. Walking with spreading legs, walking fast. 4. Walking fast changing direction, passing obstacle. 5. Sitting up and standing up, crossed arms on the chest. 6. Standing with lift the heel. 7. Hang a wooden board on the legs to load weight, increasing the weight. 8. Sitting up and standing up, putting the back on the wall	40∼60 min, 3 times/wk for 8 wks	Usual care	UPDRS I UPDRS II UPDRS III	N/A

4. Aerobic exercise						
Duncan, 2012 [32]	62 (32/30) 5.8 ± 1.1/7.0 ± 1.0 2.6 ± 0.1/2.5 ± 0.1	A: dance both leader and follower roles, change partners frequently, and learn new steps and/or integrated previously learned steps in new ways at each class throughout the 12 months	1 hr, 2 times/wk for 12 months	Usual care	MDS-UPDRS III	N/A
Hashimoto, 2015 [33]	59 (19/21/19) 6.3 ± 4.6/7.8 ± 6.2/6.9 ± 4.0 2–4 (all participants)	A: combinations of steps and movements from aerobic, jazz, and tango dances and movements from classical ballet	60 min, 1 time/wk for 12 wks	C1: Parkinson disease exercise 2 hr, 1 time/wk for 12 wks. C2: usual care	TUGT BBS total UPDRS	N/A
Ma, 2011 [34]	33 (17/16) 5.32 ± 4.43/5.16 ± 3.43 2-3 (all participants)	A: virtual reality training. Reaching for 60 fast-moving balls with the right hand	10 min training (once)	Placebo exercise. (Turning wooden cylinders with their nondominent hand)	FS	Fatigue
Romenets, 2015 [35]	33 (18/15) 5.5 ± 4.4/7.7 ± 4.7 1.7 ± 0.6/2.0 ± 0.5	A: traditional Argentine tango. Each class consisted of a review of the previous class, plus a new step or elements, followed by improvisation activities	1 hr, 2 times/wk for 12 wks	Usual care	MDS-UPDRS III TUGT	Falling, respiratory infection (unrelated with training) and fatigue

5. Complex exercise						
Ashburn, 2007 [36]	130 (65/65) 7.7 ± 5.8/9.0 ± 5.8 2–4 (all participants)	S: muscle strengthening (knee and hip extensors and hip abductors), range of movement (ankle, pelvic tilt, trunk, and head). B: static, dynamic and functional. W: inside and outside. A: U/A	60 min, daily for 6 wks	Usual care	BBS	N/A
Frazzita, 2014 [37]	25 (15/10) N/A N/A	W/B: balance and gait using a stabilometric platform with a visual cue and treadmill. All treadmill therapies were aerobic with a heart rate reserve ≤ 60% and a maximum speed of treadmill scrolling of 3.5 km/h. A: occupational therapy: transferring from sitting to standing position, rolling from supine to sitting position and from sitting to supine, dressing, use of tools, and exercises to improve hand functionality and skills. S: U/A	3 hr, 5 times/wk for 4 wks	Usual care	UPDRS III	N/A
Goodwin, 2011 [38]	130 (64/66) 9.1 ± 6.4/8.2 ± 6.4 2.6 ± 0.9/2.4 ± 0.9	B: side steps, side taps, side sway, lunges, toe walk, heel walk, and tandem walk. S: heel raise, toe raise, sit to stand, seated leg press with band, seated upper back strengthener with band, and seated outer leg strengthener with band. W: U/A A: U/A	60 min, 1 time/wk for 10 wks	Usual care	BBS TUGT	No adverse events
Ma, 2006 [39]	50 (25/25) 3.6 ± 1.8/3.5 ± 2.0 3.3 ± 0.4/3.3 ± 0.3	S: breathing training, spinal joint distraction training, changing of position, sitting up, and standing up. B: standing balance training, trunk anteflexion, extension, lateroflexion, rotation, keeping the balance during perturbed by shoulder pulls from the trainer, using weight loss device, using visual disturbance device, and standing on one foot. W: walking, walking position correcting training, and whole body training. A: U/A	30 min, 5 times/wk for 3-4 wks	Usual care	BBS TUGT	N/A
Zhang, 2013 [40]	60 (30/30) 5 Mon-6 yr (all participants) 1–5 (all participants)	S: stretching of range of motion and strengthening training (joint distraction). B: walking posture correction practice, and balance training. W: standing up training and walking training. A: U/A	8 wks	Usual care	UPDRS	N/A

E, exercise group; C, control group; H&Y, Hoehn and Yahr scale; W, walking exercise; B, balance exercise; S, strength and flexibility exercise; A, aerobic exercise; UPDRS, the Unified Parkinson's Disease Rating Scale; BBS, Berg Balance Scale; TUGT, Timed Up and Go Test; PS, preferred speed (m/s), walking on preferred velocity; FS, fast speed (m/s), walking on peak velocity; Wk, weeks; 6 MWT, 6-minute-walking-test; U/A, unapplicable; N/A, no answer.

**Table 4:** Proposal of exercise therapies for Parkinson's disease patients based on meta-analysis results.

	Walking	Strength and flexibility	Balancing	Aerobic	Complex
Motor symptoms	Recommend		Recommend	Recommend	Recommend
Nonmotor symptoms					
ADLs related to motor symptoms	Recommend	Recommend			
ADLs related to nonmotor symptoms					
Balance function		Recommend		Recommend	
Gait function			Recommend		

ADLs; activities of daily life.

## Data Availability

Data can be obtained from the corresponding author on request.
